# *Mycobacterium marinum* infection: a case report

**DOI:** 10.1186/s40409-015-0008-9

**Published:** 2015-03-20

**Authors:** Christiane Salgado Sette, Patrick Alexander Wachholz, Paula Yoshiko Masuda, Renata Borges Fortes da Costa Figueira, Fernanda Rodrigues de Oliveira Mattar, Deise Godoy Ura

**Affiliations:** Instituto Lauro de Souza Lima, Rodovia Comandante João Ribeiro de Barros, km 225/226, Bauru, SP CEP 17.034-971 Brazil; Department of Public Health, Botucatu Medical School, UNESP – Univ Estadual Paulista, Av. Prof. Montenegro Bairro: Distrito de Rubião Junior, s/n - 18618970 Botucatu, SP Brazil

**Keywords:** Mycobacterium infections, *Mycobacterium marinum*, Case report, Granuloma

## Abstract

The infection by *Mycobacterium marinum* in humans is relatively uncommon. When it occurs, it mainly affects the skin, usually with a chronic, indolent and benign evolution. The diagnosis requires a high index of suspicion, and a significant delay may be observed between the first symptoms to the final diagnosis. This present case reports a *M. marinum* infection in an immunocompetent patient that had a chronic undiagnosed injury on the dominant hand for at least five years. The patient had several medical consultations, without proper suspicion, hampering adequate diagnostic investigation. Histopathology detected tuberculoid granulomas, but showed no acid-fast bacilli. The culture in appropriate medium and the polymerase chain reaction-restriction enzyme analysis (PRA)-hsp65 confirmed the diagnosis. Treatment with clarithromycin (1 g/day) for three months was effective. Although uncommon, this infection is a contact zoonosis. Therefore, it is important for clinicians to be aware of this diagnosis and properly guide preventable measures to professionals that are in risk group.

## Background

Chronic skin lesions, especially on the extremities, are often a diagnostic challenge. The importance of a meticulous clinical investigation involves not only the proper selection of complementary tests, but also detailed anamnesis. Sometimes, important clues for diagnosis are only revealed after a thorough clinical examination and a review of occupational and/or background exposure to potential pathogens and microorganisms during foreign travels and leisure activities.

The infection by *Mycobacterium marinum* in humans – also known as aquarium granuloma, swimming pool granuloma or fish tank granuloma [1] – is an uncommon disease that mainly affects the skin, usually with a chronic, indolent and benign evolution [1]. The manifestations include granulomatous lesions, predominantly with acral distribution, that affect patients regardless of their immune status. The clinical and histopathological findings are non-specific, represented by papules, nodules or erythematous plaques, and by the presence of tuberculous granulomas with unusual evidence of bacilli on examination [1-3]. Due o diagnostic difficulties, ranging from the infrequent distribution, and the plurality of clinical presentations, the *Mycobacterium marinum* can cause from erythematous to plate-shaped, papules, nodules, single or multiple ulcerations and even sporotrichosis-like presentations, differential diagnosis with other granulomatous lesions is essential [4].

When there is clinical suspicion of *M. marinum* infection, it is mandatory to proceed to a biopsy, histopathology analysis and tissue culture. Common antibiotics are usually effective [1,5]. Herein, we report the case of an immunocompetent patient who had a chronic undiagnosed injury for at least five years, on the fifth finger of the right hand, whose careful investigation of history of exposure allowed the correct etiologic identification of *M. marinum*, and therefore the institution of proper treatment.

## Case presentation

A 51 year-old male patient, who worked as administrative assistant, reported an asymptomatic lesion on the fifth right finger with five years of evolution. The lesion initially presented as small papules, followed by scaling. He denied other skin/mucosal lesions, lymphadenopathy, reduced sensitivity, paresthesia or itching, as well as associated systemic manifestations.

Prior to consultation, the patient had received repeated prescriptions for topical corticosteroids in previous evaluations in different clinical centers, with no evidence of improvement. He had no comorbidities, denied exposure to chemicals or corrosive agents, and had no chronic drug use. His work activities were predominantly related to typing. During anamnesis, after insisting on patient’s history of exposure to agents, he revealed that during leisure time he used to take care of a home aquarium.

**Figure 1:**
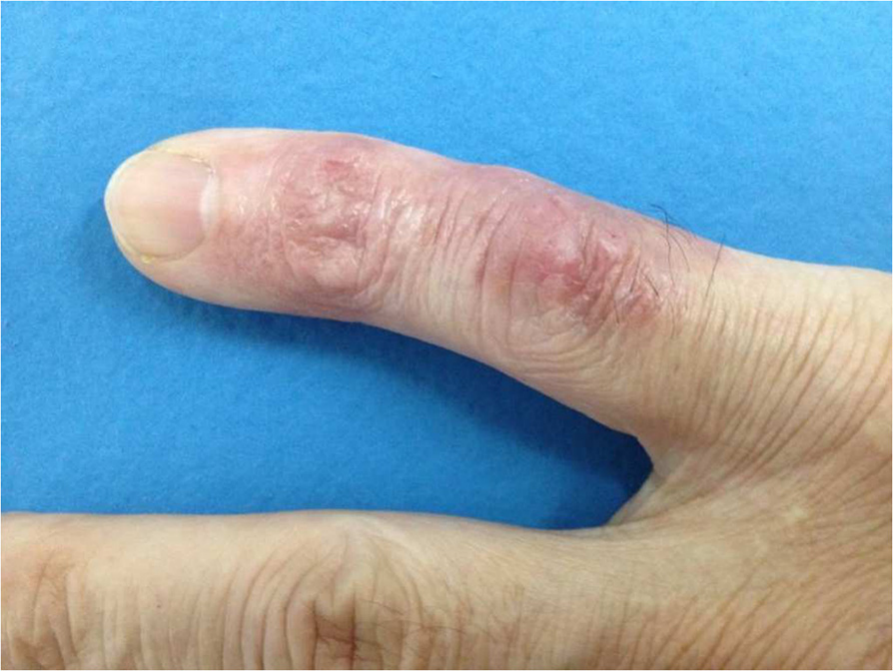
**Erythematous-brown plaque, hardened and rough, with some reddish-honey colored crusts on the dorsum of the fifth right finger, before treatment.**

On physical examination, he had a hardened and rough-brownish erythematous plaque, with a reddish-honey colored scab, on the dorsum of the fifth right finger. The lesion did not affect the nail (Figure 1). No palpable lymphnodes were found in the upper limb and/or right axillary region.

We considered mainly the following hypotheses: *Mycobacterium marinum* infection, contact dermatitis and chromomycosis. The injuries could also indicate cutaneous tuberculosis, sporotrichosis, chromoblastomycosis, leishmaniasis, verruca vulgaris, sarcoidosis, foreign body granuloma, tuberculoid leprosy, cat scratch disease, psoriasis or lichen planus hypertrophic. Thus, histopathology and tissue culture were fundamental to diagnosis accuracy.

The aquarium granuloma is an uncommon and underreported disease. Its estimated incidence is 0.04 to 0.27 per 100.000 inhabitants [4]. It belongs to a group of atypical mycobacteriosis that are caused by acid-fast bacilli (AFB), excluding *M. tuberculosis* and *M. leprae. M. marinum* was first described in 1926 as the cause of death of marine fish in an aquarium in Philadelphia (USA). In 1951, it was recognized as the pathogen responsible for human cutaneous lesions of swimmers in Sweden [4,6].

*M. marinum* is an environmental opportunistic mycobacteria that produces yellow pigments when exposed to light (photochromogenic) in appropriate medium cultures. It has a slow growing (between 2 and 8 weeks) at temperatures ranging from 30°C to 37°C (86°F to 98.60°F), and lives in aquatic environments, especially in salt water and aquariums or pools [4,6]. This mycobacterium can infect cold-blooded animals like turtles, amphibians and snakes, causing chronic systemic infection in fish [1,6]. Dead fish can serve, as well, as reservoirs [4,7].

Occasionally, the pathogen causes granulomatous lesions on the human skin. The disease occurs more frequently in individuals who are exposed to aquatic environments through labor occupation or leisure activities [4]. The infection has no gender predilection, and predominates in the second and third decades of life, the period of greatest occupational exposure [4,7,8].

*M. marinum* usually develops after minor trauma or contact with fish and/or their reservoirs [1,6]. The incidence is similar between immunocompetent and immunocompromised patients, but the clinical outcomes are different [4,7]. It usually develops erythematous nodules at the inoculation site, with a rough and sometimes verrucous surface, that may become a plaque and ulcerate, or follow the lymphatic path in a sporotrichosis-like aspect [4,7,8]. The course of disease is indolent, with cases of spontaneous healing in a immunocompetent person reported after two to three years of evolution [6,9]. Rarely, adjacent structures such as bones and joints are affected, causing osteomyelitis, tenosynovitis, bursitis and arthritis [6].

**Figure 2:**
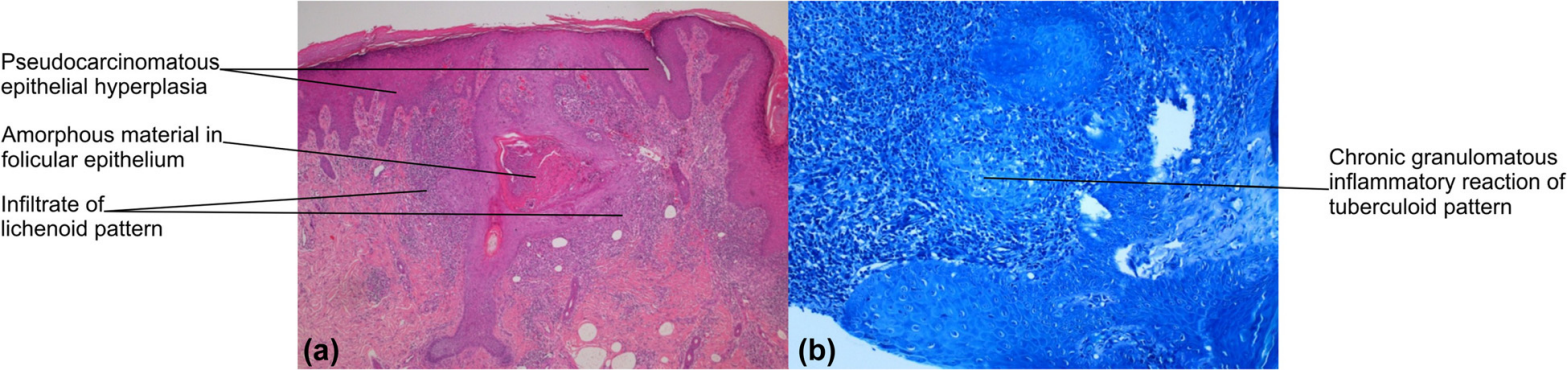
**Skin biopsy - Histopathology. (a)** Pseudocarcinomatous epithelial hyperplasia with amorphous material in the follicular epithelium, which is surrounded by intense infiltrates of lichenoid pattern (HE, original magnification 40×). **(b)** Chronic granulomatous inflammatory reaction of tuberculoid pattern with focus of fibrinoid necrosis and absence of acid-fast bacilli (Fite-Faraco, original magnification 200×).

In the present case report, we performed a biopsy, which revealed pseudocarcinomatous epithelial hyperplasia with chronic granulomatous inflammatory reaction of tuberculoid pattern, with focus of fibrinoid necrosis and absence of acid-fast bacilli (HE and Fite-Faraco stain) (Figure 2).

Histopathological findings of mycobacteriosis commonly consist of a suppurative and granulomatous process in the dermis, with parakeratosis, acanthosis and ulceration in the epidermis [10]. Pseudocarcinomatous hyperplasia may also occur. Only in a few cases bacilli are seen, even with special stains such as Periodic acid-Schiff stain (PAS) and Fite-Faraco, exceptionally in immunocompromised patients [10]. Granulomatous inflammation is most frequently found in *M. marinum* infections than other non-tuberculous mycobacteria; the caseation is absent, but there fibrinoid necrosis [10]. Thus, confirmation is usually obtained by culture and polymerase chain reaction (PCR) test [10].

**Figure 3:**
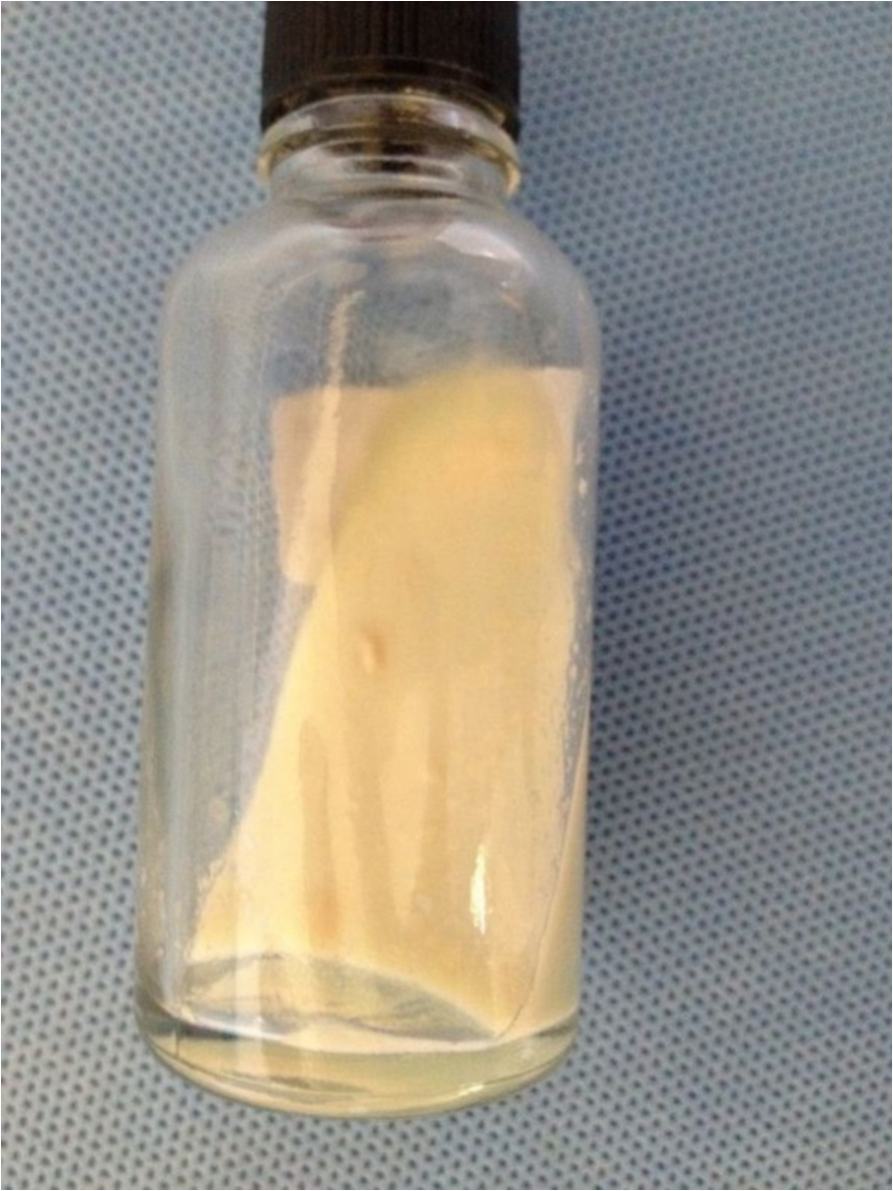
***Mycobacterium marinum*** **culture on Löwenstein-Jensen medium, after 12 days of incubation at 26°C (78.800°F).**

In the present case, the mycobacterial culture in Löwenstein-Jensen medium was positive for *M. marinum* (Figure 3), and PCR-restriction enzyme analysis (PRA) of hsp65 was indicative of *M. marinum* infection [11].

Diagnosis of this infection is suspected mainly by clinical history, occupational backgrounds and lifestyle habits. Although histopathology is important in the differential diagnosis, the confirmation is performed through culture on Löwenstein-Jensen medium [1,8]. Emerging studies have demonstrated that PCR can become a fast, sensitive and specific diagnostic tool, concerning a more comprehensive method. However, it should be interpreted with caution, as false-positives are possible [7,9]. The PRA-hsp65 is a rapid and highly reliable method in the identification of non-tuberculous mycobacteria. In this molecular method, a fragment of the hsp65 gene is amplified by PCR and then analyzed by restriction digest; this rapid approach offers the promise of accurate, cost-effective species identification [11].

**Figure 4:**
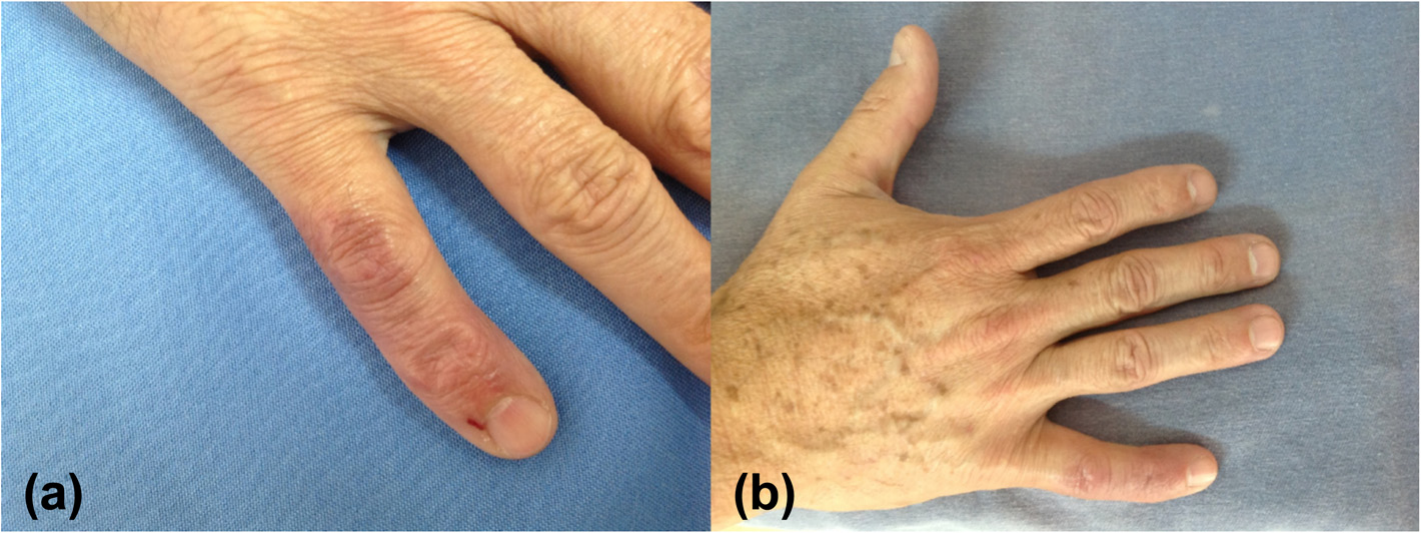
**Evolution during and after treatment. (a)** Dorsum of the fifth right finger, one month after the beginning of treatment**. (b)** Dorsum of the right finger, three months after initiation of treatment.

After being diagnosed, the patient was treated with clarithromycin (1 g/day) for three months, resulting in regression of the lesion (Figure 4). *M. marinum* is generally sensitive to multiple antibiotics [4,7,9,12]. Nonetheless, due to the absence of better evidence, there is no standard treatment to be recommended, with proven efficacy and effectiveness. Generally, strains of *M. marinum* are susceptible to antituberculous drugs and common antibiotics (such as quinolones, tetracyclines, macrolides, aminoglycosides, etc.). In addition, monotherapy can eliminate cutaneous infections successfully. Treatment failure is usually related to deep structure involvement or inappropriate therapies [5,12].

In superficial skin infections, clarithromycin, minocycline, doxycycline and trimethoprim-sulfamethoxazole are used as monotherapy [6,9]. Ciprofloxacin and doxacycline have shown effectiveness in some reports [9,13,14]. A combined therapy with two or more drugs (e.g., rifampicin associated with ethambutol) might be required due to drug resistance [6,9].

In severe infections, including those with a sporotrichosis-like distribution, an isolated combination of rifampicin and ethambutol has been recommended [6,9]. In cases of osteomyelitis and/or associated arthritis, some authors suggest treatment with clarithromycin and ethambutol, with the possible addition of rifampicin [4,6,9]. Other researchers propose the addition of levofloxacin when there is suspicion of other atypical infections, or in cases of intolerance and/or allergy to first choice drugs [7,15].

Treatment should be administered for at least six weeks up to 12 months, depending on the clinical evolution of the lesion [12]. In unresponsive cases, amikacin may be a good option, prescribed in low doses, to reduce the risks of adverse effects [16].

Resection and debridement of the lesion are generally not recommended, and are only indicated in cases refractory to treatment with antibiotics [8,9]. Some studies showed worsening of the condition if it is carried out [17]. Apparently, the intervention may be indicated as an adjunctive treatment in cases of tissue necrosis and septic arthritis, facilitating the effects of the antibiotics [12]. Cryotherapy, laser and photodynamic therapy have been reported as effective treatment alternatives, but there are few studies evaluating the efficiency of these methods [6,9].

We have chosen to start treatment with clarithromycin, since the patient was healthy, immunocompetent, cognitively capable, and with a well-localized lesion. We followed the progress with monthly outpatient visits. He had a good clinical response with monotherapy: the lesion showed involution in the second month of treatment.

## Conclusions

In summary, the present report describes a case of *M. marinum* infection in an immunocompetent patient, with a lesion on the dominant hand, with five years of evolution. The patient had several medical consultations, without proper suspicion due to the lack of adequate investigation of his habits and exposures in leisure activity, hampering adequate diagnostic investigation. Histopathology detected tuberculoid granulomas, but showed no acid-fast bacilli. The culture in appropriate medium and the PCR test confirmed the diagnosis; treatment with clarithromycin (1 g/day) for three months was effective.

*M. marinum* is considered an uncommon cause of skin infections in humans. The diagnosis requires a high index of suspicion; therefore, significant delay may be observed between first symptoms and diagnosis confirmation. This infection should be included in the differential diagnosis of chronic wound cases with difficult diagnosis in the upper extremities, especially if a history of exposure to aquariums or handling fish is identified [8,9].

Since this infection is a contact zoonosis, it is important for clinicians to be aware of its diagnosis and properly guide professionals that are in risk groups (such as aquaculture specialists, fishery workers, ornamental fish hobbyists) that this infection can be prevented by the use of waterproof gloves.

## Consent

Written informed consent was obtained from the patient for publication of this case report and any accompanying images.

## Abbreviations

AFB: Acid-fast bacilli
PAS: Periodic acid-Schiff stain
PCR: Polymerase chain reaction

## References

[1] Slany M, Jezek J, Bodnarova M (2013). Fish tank granuloma caused by *Mycobacterium marinum* in two aquarists: two case reports. Biomed Res Int.

[2] Palamaras I, Pietropaolo N, El-Jabbour J, Thomson P, Dissanayake M, Robles W (2008). Axonal sensory neuropathy in a patient treated with minocycline for fish-tank granuloma. J Eur Acad Dermatol Venereol.

[3] Ramos JM, García-Sepulcre MF, Rodríguez JC, Padilla S, Gutiérrez F (2010). *Mycobacterium marinum* infection complicated by anti-tumour necrosis factor therapy. J Med Microbiol.

[4] Jaled MM, Pedrini Cinqualbrez MF, González P, Förster Fernández J, Anaya JS, Stengel FM (2010). Infección por *Mycobacterium marinum*. Características epidemiológicas, clínicas y tratamiento. Med Cutan Iber Lat Am.

[5] Huang Y, Xu X, Liu Y, Wu K, Zhang W, Liu P (2012). Successful treatment of refractory cutaneous infection caused by *Mycobacterium marinum* with a combined regimen containing amikacin. Clin Interv Aging.

[6] García Acebes CR, Barchino Ortiz L, Aboín González S, Díaz Ley B, Ruiz Fernández P, Sánchez de Paz F (2006). Infección por *Mycobacterium marinum.* Presentación de un nuevo caso y revisión de la literatura. Actas Dermosifiliogr.

[7] Ang P, Rattana-Apiromyakij N, Goh CL (2000). Retrospective study of *Mycobacterium marinum* skin infections. Int J Dermatol.

[8] Cheung JP, Fung B, Ip WY, Chow SP (2012). *Mycobacterium marinum* infection of the hand and wrist. J Orthop Surg (Hong Kong).

[9] Rallis E, Koumantaki-Mathioudaki E (2007). Treatment of *Mycobacterium marinum* cutaneous infections. Expert Opin Pharmacother.

[10] Calonje JE, Brenn T, Lazar AJ, Mckee PH. (Editors): McKee´s pathology of the skin. 4th edition. Boston: Saunders; 2011.

[11] Chimara E, Ferrazoli L, Ueky SYM, Martins MC, Durham AM, Arbeit RD (2008). Reliable identification of mycobacterial species by PCR-restriction enzyme analysis (PRA)-*hsp*65 in a reference laboratory and elaboration of a sequence-based extended algorithm of PRA-*hsp*65 patterns. BMC Microbiol.

[12] Flondell M, Ornstein K, Björkman A (2013). Invasive *Mycobacterium marinum* infection of the hand. J Plast Surg Hand Surg.

[13] Aubry A, Chosidow O, Caumes E, Robert J, Cambau E (2002). Sixty-three cases of *Mycobacterium marinum* infection: clinical features, treatment, and antibiotic susceptibility of causative isolates. Arch Intern Med.

[14] Petrini B (2006). *Mycobacterium marinum*: ubiquitous agent of waterborne granulomatous skin infections. Eur J Clin Microbiol Infect Dis.

[15] Griffith DE (2007). Therapy of nontuberculous mycobacterial disease. Curr Opin Infect Dis.

[16] Dodiuk-Gad R, Dyachenko P, Ziv M, Shani-Adir A, Oren Y, Mendelovici S (2007). Nontuberculous mycobacterial infections of the skin: a retrospective study of 25 cases. J Am Acad Dermatol.

[17] Chow SP, Ip FK, Lau JH, Collins RJ, Luk KD, So YC (1987). *Mycobacterium marinum* infection of the hand and wrist. Results of conservative treatment in twenty-four cases. J Bone Joint Surg Am.

